# Machine learning to predict adverse drug events based on electronic health records: a systematic review and meta-analysis

**DOI:** 10.1177/03000605241302304

**Published:** 2024-12-13

**Authors:** Qiaozhi Hu, Jiafeng Li, Xiaoqi Li, Dan Zou, Ting Xu, Zhiyao He

**Affiliations:** 1 Department of Pharmacy, West China Hospital, Sichuan University, Chengdu, Sichuan, China; 2 West China School of Medicine, 12530 Sichuan University, Chengdu, Sichuan, China; 3 Mental Health Center, West China Hospital, Sichuan University, Chengdu, Sichuan, China; 4 Key Laboratory of Drug-Targeting and Drug Delivery System of the Education Ministry, Sichuan Engineering Laboratory for Plant-Sourced Drug and Sichuan Research Center for Drug Precision Industrial Technology, West China School of Pharmacy, Sichuan University, Chengdu, Sichuan, China

**Keywords:** Meta-analysis, machine learning, adverse drug event, prediction model, electronic health record, systematic review

## Abstract

**Objective:**

This systematic review aimed to provide a comprehensive overview of the application of machine learning (ML) in predicting multiple adverse drug events (ADEs) using electronic health record (EHR) data.

**Methods:**

Systematic searches were conducted using PubMed, Web of Science, Embase, and IEEE Xplore from database inception until 21 November 2023. Studies that developed ML models for predicting multiple ADEs based on EHR data were included.

**Results:**

Ten studies met the inclusion criteria. Twenty ML methods were reported, most commonly random forest (RF, n = 9), followed by AdaBoost (n = 4), eXtreme Gradient Boosting (n = 3), and support vector machine (n = 3). The mean area under the summary receiver operator characteristics curve (AUC) was 0.76 (95% confidence interval [CI] = 0.26–0.95). RF combined with resampling-based approaches achieved high AUCs (0.9448–0.9457). The common risk factors of ADEs included the length of hospital stay, number of prescribed drugs, and admission type. The pooled estimated AUC was 0.72 (95% CI = 0.68–0.75).

**Conclusions:**

Future studies should adhere to more rigorous reporting standards and consider new ML methods to facilitate the application of ML models in clinical practice.

## Introduction

Drug therapy carries potential risks of medication-related injuries, which are associated with notably prolonged hospital stays, increased economic burdens, and a nearly 2-fold higher risk of mortality.^1^ Adverse drug events (ADEs) represent a global public health challenge that affects patients and healthcare systems.^2^ ADEs are drug-related patient injuries caused during the drug use process that could potentially be prevented.^3^ Therefore, numerous studies have concentrated on predicting ADEs.

Researchers have focused on ADE prediction based on various factors, including drug–drug interactions (DDIs),^3^ the chemical structures of drugs,^4^ spontaneous reporting systems, and health records.^5^ DDIs can manifest in various ways, including effects on pharmacokinetics and pharmacodynamics, resulting in altered drug concentrations or increased susceptibility to organ toxicity.^6,7^ Therefore, DDIs can potentially elicit adverse effects in patients, leading to significant consequences.^8^ The chemical composition of a drug possesses inherent structural characteristics that can influence its propensity to induce adverse effects.^9^ However, these approaches do not provide patients information, such as age, sex, length of stay, total dose, and diagnosis. Spontaneous reporting systems can provide some patient information, whereas other information, such as the length of stay, dose per patient, and the total number of patients using a certain medication, remain unavailable.^10^

Health records contain comprehensive data spanning the entire duration of patients’ hospitalization. Using predictive tools for ADE anticipation in hospitalized patients has the capacity to assist clinicians in proactively averting ADEs at the individual patient level.^11^ Furthermore, the elucidation of patterns underlying the occurrence of ADEs during hospitalization, as derived through this approach, can inform the selection of interventions to enhance medication safety and ultimately reduce the incidence of ADEs.

With the burgeoning popularity of electronic health record (EHR) systems, it becomes increasingly feasible to detect and predict drug-related injuries. EHRs can help detect potential ADEs,^12^ representing an appealing alternative to the arduous manual review of patient charts. Subsequently, adverse reactions can be predicted through the application of statistical models. Therefore, an escalating number of studies have focused on the prediction of ADEs using EHR data.^13^ The intricate nature of drug-related injuries in clinical practice often entails the compounding effects of multiple medications, with certain medications evincing the capacity to induce multiple concurrent or sequential adverse events. Therefore, predictive studies targeting multiple adverse events are more aligned with clinical imperatives, and they carry practical significance. However, the prediction of multiple adverse events involves more risk factors, which can lead to the unsatisfactory performance of traditional statistical methods. For example, logistic regression (LR) is a conventional statistical method commonly used to predict ADE, but its F1 score is only 17% to 36%.^1,14,15^

Machine learning (ML), situated within the domain of artificial intelligence, represents an interdisciplinary field encompassing statistics, computer science, and various ancillary domains. ML exhibits proficiency in managing intricate non-linear associations between variables and outcomes, affording heightened generalization capabilities and augmented precision.^16^ ML has emerged as a burgeoning focal point in medical applications, demonstrating substantial potential in disease diagnosis,^17^ prescription analysis,^18^ and complications surveillance.^19^ Many studies have applied ML to predict multiple ADEs, but there is a lack of relevant research evaluating these studies systematically. Therefore, we conducted a systematic review to provide a comprehensive overview of the application of ML in predicting multiple ADEs based on EHR data. The corresponding protocol has been posted on the Research Square preprint platform.^20^

## Materials and methods

This systematic review was conducted in accordance with the Preferred Reporting Items for Systematic Reviews and Meta-Analyses (PRISMA) guidelines.^21^ The details of the PRISMA guidelines are provided in Table S1. The review protocol was formally registered as a systematic review with PROSPERO under the registration number CRD42023464771. PROSPERO is funded by the United Kingdom National Institute for Health Research. The PROSPERO register accepts any systematic review, and its registration process is simple and capable of providing strong evidence for evidence-based decision-making to facilitate systematic review of research.^22^

### Search strategy and selection criteria

Four electronic databases, namely PubMed, Web of Science, Embase, and IEEE Xplore, were searched by title and abstract from each database’s date of inception to 21 November 2023. The search strategy involved the use of MeSH and Emtree keywords, as well as their synonyms and keywords obtained from the initially reviewed papers. The search strategy used for each individual database is provided in Table S2.

The inclusion criteria were as follows: focused on the prediction of multiple ADEs, applied ML algorithms based on EHRs, and provided sufficient explanations regarding the research findings. Studies were excluded if they met any of the following criteria: lacking a full-text paper, review articles, written in languages other than English, focused on medical safety events, concentrated on the identification of ADEs, used conventional algorithms such as logistic regression, and not based on EHRs.

### Study selection and data extraction

After removing duplicate studies, two independent reviewers (QZ Hu and CQ Li) independently evaluated the titles and abstracts retrieved in the searches. The reviewers have systematic review experience, and they received training prior to this study to avoid the deletion of important studies. The full text of potentially eligible studies was assessed to confirm their suitability. Any discordant evaluations were resolved through consensus.

Study information was extracted, including the author, year, study setting, demographic characteristics, duration of data collection, used ML algorithms, performance metrics (such as accuracy, sensitivity, specificity, precision, and F1 score), and risk factors.

### Quality evaluation

Two assessment instruments were employed to assess the quality of the included articles. Predictive models in healthcare use predictors to estimate the probability of an individual developing a condition or disease in the future. The Prediction Model Risk of Bias Assessment Tool (PROBAST) is a tool for assessing the risk of bias and the applicability of prediction model studies, and it can be used as a guide to systematically evaluate the quality of papers related to modeling and prediction, evaluate the quality of papers, and assess the risk of bias.^23^ PROBAST consists of 20 signaling questions across four domains: participants, predictors, outcomes, and analysis. The applicability of the study within the first three domains was also scrutinized and categorized as low, high, or unclear.^23^

The quality of artificial intelligence studies in the medical field was assessed by implementing the Checklist for the Assessment of Medical AI (ChAMAI).^24^ The original purpose of the tool is to help editors and reviewers discriminate between high-quality contributions and manuscripts that should be rejected.^24^ ChAMAI comprises 30 items distributed across six dimensions, namely problem understanding, data understanding, data preparation, modeling, validation, and deployment.^24^ These items can be divided to low- and high-priority types, and they were evaluated as OK (adequately addressed), mR (sufficient but improvable), or MR (inadequately addressed).^24^ For high-priority items, scores of 0, 1, and 2 were assigned for OK, mR, and MR, respectively, whereas for low-priority projects, the scores were halved (i.e., 0, 0.5, and 1).^25^ The maximum score for this assessment tool was 50 points.

### Statistical analysis

The effects and 95% confidence intervals (95% CIs) were examined using a random-effects model.^26^ Pooled sensitivity and specificity and their respective 95% CIs were calculated using contingency tables. The overall performance of ML was evaluated using the summary receiver operator characteristics (SROC) curve and the area under the SROC curve (AUC). Statistical significance was signified by *P < *0.05. Heterogeneity was assessed using the Q and *I*^2^ statistics, with *I*^2^ > 50% indicating significant heterogeneity. If heterogeneity was significant, then the random-effects model was used to pool the effect estimates and assess potential sources of heterogeneity. Publication bias was evaluated using the funnel plot and regression test.^26^ All statistical analyses were conducted using Stata 16.0 software (StataCorp, College Station, TX, USA).^27^

## Results

### Characteristics of the included studies

**Figure 1.**
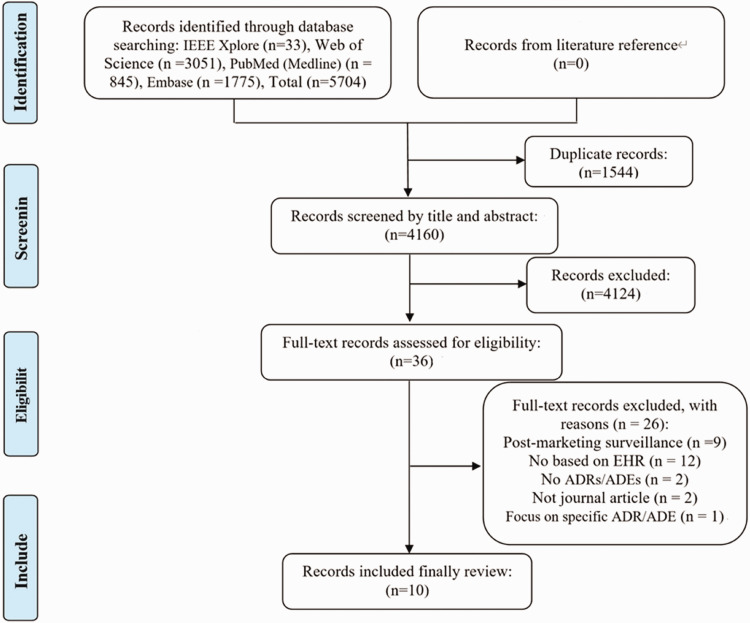
PRISMA flow diagram of the citation search and selection strategy. ADE, adverse drug event; EHR, electronic health record; PRISMA, Preferred Reporting Items for Systematic reviews and Meta-Analyses.

The initial database search yielded 5704 relevant studies. After removing duplicates, the titles and abstracts were screened for relevance. The full text of 36 studies was assessed, and 10 studies^5,15,28–35^ met the inclusion criteria, as presented in Figure 1.

These studies included three studies based on self-built databases,^5,30,35^ one study based on the Medical Information Mart for Intensive Care (MIMIC)-IV (version 2.1),^15^ one based on a Chinese EMR database,^35^ and five studies based on the Stockholm Electronic Patient Record (EPR) Corpus.^28,29,31–33^ These studies were conducted in China,^5,30,34^ the United States,^15^ India,^29^ and Sweden,^28,29,31–33^ and they covered a range of targeted populations, including older adults,^5^ pediatric patients,^30^ and the general population.^15,28,29,31–35^

These studies frequently employed multiple ML algorithms simultaneously to compare the performance of different models on the same dataset. Among the 20 ML methods documented across the included studies, random forest (RF) was most frequently used (n = 9), followed by Adaboost (n = 4), eXtreme Gradient Boosting (XGBoost, n = 3), and support vector machine (SVM, n = 3). Four studies improved the ML algorithms by adjusting their configurations, learned weights, or tree sizes to optimize model performance.^28,29,31,33^ Detailed information is presented in Table 1.

**Table 1.** Characteristics of the included studies.

Study ID	No. of included patients	Data period	Data types	Age	Male (%)	No. of patients with ADE (%)	No of patients with ADE	No. of drugs that cause ADE	Length of stay
Hu, 2022^5^	1800	2015–2017	Structured clinical data	Total: 69.84 ± 8.14No ADE: 70.12 ± 8.19ADE: 67.95 ± 7.55	58.56%	234 (13.00%)	296	21	Total: 10.19 ± 8.94 No ADEs : 9.44 ± 8.07ADEs :15.19 ± 12.28
Yu, 2021^30^	1746	2013–2015	Structured clinical data	Total: 3.84 ± 3.89No ADE: 3.86 ± 3.85ADE: 3.72 ± 4.12	Total: 65.00%No ADE: 64.70%ADE: 67.40%	221 (12.70%)	247	27	Total: 7.83 ± 5.29No ADE: 7.48 ± 4.66ADE: 10.23 ± 8.03
Langenberger, 2023^15^	210,181	2008–2019	Unstructured and structured clinical data	No ADE: 59.8 ± 19.7ADE: 63.3 ± 16.9	No ADE: 49.40%ADE: 51.50%	10,957 (5.21%)	22,667	N	No ADE: 4.00 ± 6.49ADE: 9.12 ± 11.8
Karlsson, 2014^28^	16,287	2009–2010	Unstructured and structured clinical data	N	N	4128 (25.34%)	N	N	N
Ponraj, 2021^35^	5000	N	Structured clinical data	N	N	N	N	N	N
Karlsson, 2016^29^	35,711	2007–2014	Unstructured and structured clinical data	N	N	16,062 (44.98%)	N	N	N
Zhao, 2021^34^	30,703	N	Unstructured and structured clinical data	N	N	N	N	N	N
Zhao, 2015a^32^	14,303	2009–2010	Unstructured and structured clinical data	N	N	2807 (19.62%)	N	N	N
Zhao, 2015b^33^	14696	2009–2010	Unstructured and structured clinical data	N	N	2928 (19.92%)	N	N	N
Zhao, 2016^31^	38,709	2009–2015	Unstructured and structured clinical data	N	N	5733 (14.81%)	N	N	N

The top five types of drugs that cause ADEs (n)	Type of ADE	The top five types of ADEs (n)	Important risk factors	Machine learning algorithms	Evaluation and validation	Evaluation indicator	Optimal model performance
Antineoplastic (165), antimicrobial (44), anticoagulant (18), antihypertensive (13), analgesic (9)	32	Nausea (64), leukopenia (36), hepatotoxicity/transaminase disorder (25), diarrhea (22), vomiting (22)	Number of true triggers (+), length of stay, doses per patient, age, number of admissions in the previous year, surgery, drugs per patient, number of medical diagnoses, antibacterial use, and sex	Seven algorithms: XGBoost, AdaBoost, CatBoost, GBDT, LightGBM, TPOT, and RF	Training and testing	AUC, accuracy, precision, recall, F1 score, precision–recall curve	AdaBoost with the best predicting abilities (accuracy: 88.06%, precision: 68.57%, recall: 48.21%, F1 score: 52.75%, AUC: 0.91)
Antibacterials (86), sedatives (55), immunomodulators (14), antiepileptics (12), potassium chloride, glucose (10)	28	Oversedation\hypotension (47), rash (35), diarrhea (34), candidiasis (25), constipation (21), vomiting (21)	Number of true triggers (+), number of doses, BMI, number of drugs, number of admissions, height, length of hospital stays, weight, age, and number of diagnoses	Seven algorithms: XGBoost, AdaBoost, CatBoost, GBDT, LightGBM, TPOT, and RF	Training and Testing	AUC, precision, recall, F1 score, precision–recall curve	GBDT with the best predictive performance (precision: 44%, recall: 25%, F1 score: 31.88%, AUC: 0.81)
Antineoplastic (165), antimicrobial (44), anticoagulant (18), antihypertensive (13), analgesic (9)	102	Other secondary thrombocytopenia (5153), other iatrogenic hypotension (4388), fever, unspecified (4010), dermatitis attributable to drugs and medicines taken internally (1871), enterocolitis attributable to *Clostridium difficile* (1222)	Admission type, temperature, chief complaint, systolic blood pressure, age, serotonin receptor antagonists, heart rate, glucocorticoids, antiviral agents, and rifamycin	Six algorithms: RF, GBM, Ridge, LASSO, EN, and LR	Training and testing	AUC, accuracy, precision, recall, F1 scores, G-mean, Youden index, negative predictive value, specificity, precision–recall curve, Brier score	GBM with the best predictive performance (accuracy: 66.67%, precision: 10.10%, recall: 67.50%, F1 score: 17.50%, negative predictive value: 97.50%, specificity: 66.50%, G-mean: 66.30%, Youden index: 35.00%, AUC: 0.75, AUC-PR: 0.13
N	14	N	N	Four algorithms: RF with four tree sizes and four configurations [two (log and sqrt) as baseline and two re-sampling approaches (ren and reinf)]	10-fold cross-validation	AUC, F1 score, Brier score, prediction	RF-ren model had the best predictive performance (F1 score: 84.02%, AUC: 0.95, Brier score: 12.98%). However, no significant relationship was observed between the models.
N	N	N	N	Three algorithms: SVM, DT, and MLP	Training and testing	Accuracy	DT with the best performance (fatality rate accuracy: 91.40%, severity rate accuracy: 81.70%)
N	27	Allergy, unspecified (5540), unspecified adverse effect of drug or medicament (1967), angioneurotic edema (1146), maternal care for (suspected) damage to fetus by drugs (1096), Generalized skin eruption attributable to drugs and medicaments (861)	N	Five algorithms: RPF with four configurations [RPF-ed (1), RPF-ed (2), RPF (3,1), and RPF (3,2)] and RF	10-fold-cross validation	F1 score, Brier score, prediction	There was no significant difference in performance between RPF and RF (RF F1 score: 84.45%, RF Brier score: 11.66%)
N	N	N	Age, course of treatment, transfusion stash time, infusion speed, solvent dosage, solvent category, dermatitis, Xueshuantong, Naodanbai, hypertension, Sanqi Xiaozhong, capsule, fasudil, chronic obstructive pulmonary disease, loose-jointed pill, enteritis, erysipelas, syringomyelia, pelvic Infection, aescin	Six algorithms: SVM, RF, AdaBoost, XGBoost, ANN, and LR	Training and testing	Sensitivity, specificity, recall F1 scores	XGBoost, AdaBoost, and RF exhibited relatively similar performance (XGBoost recall: 75.00%, XGBoost specificity: 92.86%, XGBoost F1 score: 82.98%)
N	27	Allergy, unspecified (737), unspecified adverse effect of drug or medicament (442), Other complications following infusion, transfusion, and therapeutic injection (342), generalized skin eruption attributable to drugs and medicaments (156), angioneurotic edema (108)	N	Nine algorithms: DT, SVM Poly, SVM RBF, RF, KNN, AdaBoost, nagging, NB, and LR	Training and testing10-fold cross-validation	AUC, accuracy	RF (accuracy: 84.47%, AUC: 0.76)
N	14	Allergy, unspecified (984), unspecified adverse effect of drug or medicament (472), other complications following infusion, transfusion, and therapeutic injection (353), maternal care for (suspected) damage to fetus by drugs (334), generalized skin eruption attributable to drugs and medicaments (174)	N	Three algorithms: RF with three strategies (BE, BBE, and BWE)	Training and testing 10-fold cross-validation	AUC, accuracy, precision, recall, F1 scores, precision–recall curve	RF-BWE had the best predictive performance (accuracy: 88.35%, recall: 43.49%, precision: 52.21%, F1 score: 45.00%)
N	19	Secondary thrombocytopenia (1246), unspecified adverse effect of drug or medicament (1047), drug-induced aplastic anemia (593), allergy (574), other complications following infusion, transfusion, and therapeutic injection (538)	N	Four algorithms: RF with two learned weights and two preassigned weights (LWA, LWS, PWA, and PWS)	Training and testing10-fold cross-validation	AUC, accuracy, precision–recall curve	RF-LWS had the best predictive performance (accuracy: 88.85; AUC: 0.80)

### Evaluating the quality of studies

The biased risk assessment results of PROBAST indicated that the majority of included studies featured a high risk of bias while demonstrating a low risk of applicability concerns (Table S3 and Figure S1). Among the 10 included studies, seven were deemed to have a high risk of bias, and three were found to have a high risk of applicability concerns. Only three studies^5,15,30^ displayed both a low risk of bias and a low risk of applicability concerns.

Based on the assessment using ChAMAI, the overall mean score of the included studies was 23.7 (95% CI = 20.00–32.50), which constituted less than 50% of the maximum score (Table S4 and Figure S2). Only three studies achieved a score of at least 25.00. Low mean scores were recorded for the dimensions of data understanding, data preparation, and deployment, each falling below 50% of the maximum score. Conversely, the dimensions of problem understanding, modeling, and validation achieved high mean scores, particularly with respect to modeling, for which a full score was obtained.

### ADEs

Eight studies^5,15,28–33^ provided information on the types of ADEs identified, with the reported numbers ranging from 14 to 102 (Table S5). Drug allergies, angioneurotic edema, Stevens–Johnson syndrome (SJS), anaphylactic shock, and contact dermatitis were discussed in all eight studies. Additionally, oversedation/hypotension, hypoglycemia, thrombocytopenia, cardiomyopathy attributable to drugs and external agents, nephrotoxicity/creatinine disorder, and drug-induced adrenocortical insufficiency were commonly documented.

Convulsions, hyperglycemia, respiratory depression, bronchospasm, and dyspnea were only mentioned in the study targeting children,^30^ whereas bradycardia was only mentioned in the study of older patients.^5^ The incidences of the aforementioned ADEs were lower than 1%.^5,30^

### Predictive performance for different predictive methods

The AUC serves as a critical indicator of model performance. Seven studies reported AUCs ranging from 0.26 to 0.95.^5,15,28,30–33^ ML algorithms such as Light Gradient-Boosting Machine (LightGBM), AdaBoost, CatBoost, XGBoost, and TPOT demonstrated high AUCs exceeding 0.90. RF, with an average AUC of 0.83, exhibited the potential to achieve high AUCs when combined with resampling-based approaches, yielding a range of 0.9448 to 0.9457.

**Figure 2.**
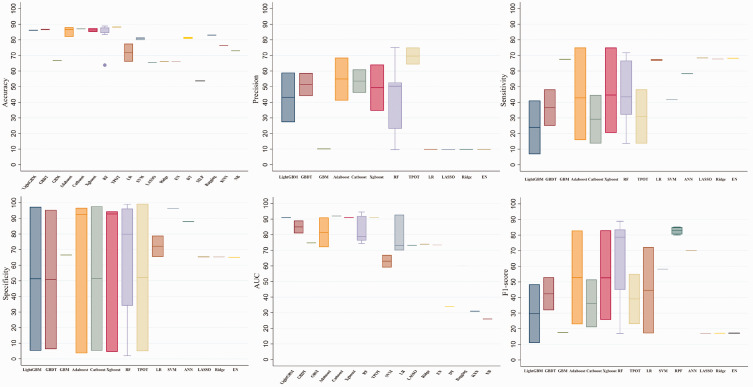
Summary of prediction model performance. XGBoost, eXtreme Gradient Boosting; GBDT, gradient boosting decision tree; LightGBM, Light Gradient-Boosting Machine; GBM, gradient boosting machine; RF, random forest; TPOT, Tree-based Pipeline Optimization Tool; LR, logistic regression; SVM, support vector machine; LASSO, least absolute shrinkage and selection operator; EN, elastic net; DT, decision tree; MLP, multilayer perceptron; KNN, K-nearest neighbor; NB, naïve Bayes; ANN, artificial neural network.

The performance was assessed using metrics such as accuracy, precision, sensitivity, specificity, and the F1 score, as reported in seven, four, five, five, and five studies, respectively. The mean accuracy, precision, sensitivity, specificity, and F1 score were 80.77%, 41.48%, 46.02%, 63.37%, and 51.16%, respectively, as presented in Figure 2.

### Meta-regression

**Figure 3.**
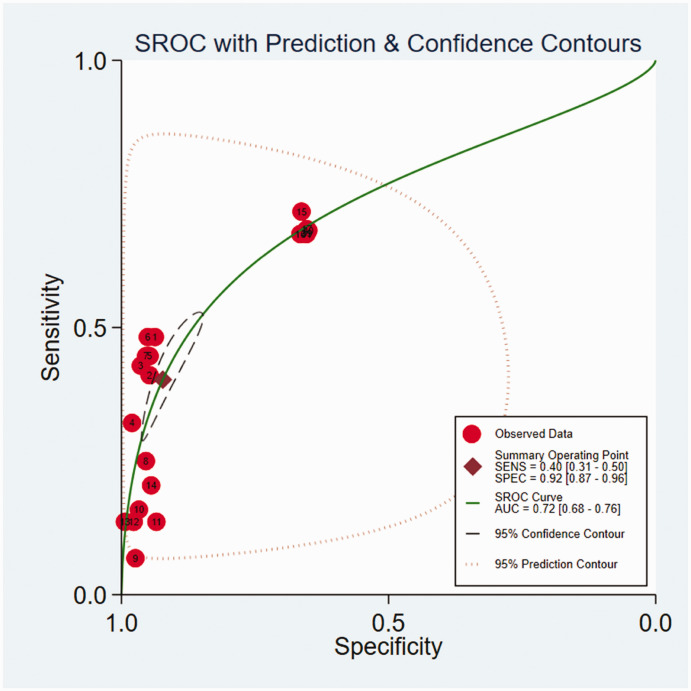
Hierarchical summary receiver operating characteristic curves of studies included in the meta-analysis to classify adverse drug effects from three studies. The 95% confidence interval is a visual representation of between-study heterogeneity.

**Figure 4.**
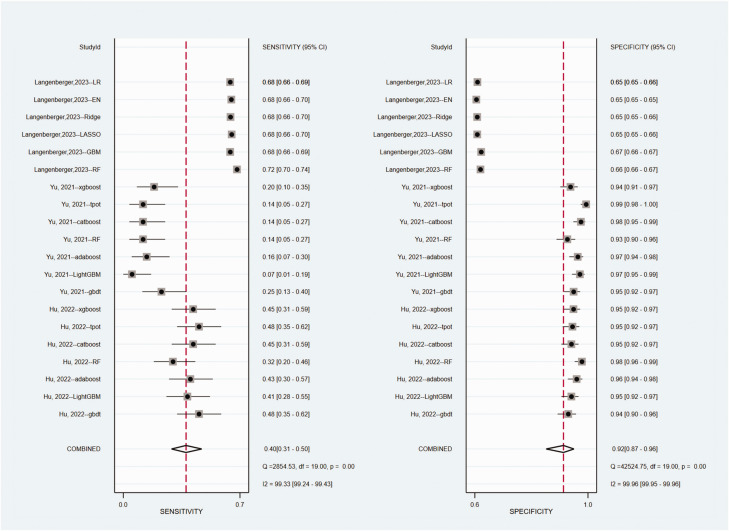
Forest plots of sensitivity and specificity.

The contingency tables from three prediction studies^5,16,30^ were extracted. The details are presented in Table S6. The pooled estimated AUC was 0.72 (95% CI = 0.68–0.75, Figure 3, whereas the pooled sensitivity and specificity were 0.40 (95% CI = 0.31–0.50, *I*^2^ = 99.33%) and 0.92 (95% CI = 0.87–0.96, *I*^2^ = 99.96%). In comparison with LR, the meta-regression analysis indicated that ML algorithms yielded higher specificity [ML vs. LR: 0.93 (95% CI = 0.88–0.96) vs. 0.65 (95% CI = 0.65–0.66)] but lower sensitivity [ML vs. LR: 0.38 (95% CI = 0.29–0.49) vs. 0.68 (95% CI = 0.66–0.69, Figures 4 and S3).

### Heterogeneity

The meta-regression results revealed substantial heterogeneity regarding the pooled estimate (*I*^2^ > 99%). The results of sensitivity analysis illustrated that the within-study heterogeneity was low. Because different ML were used in three prediction studies, we could not conduct sensitivity analysis of the different models. The results of sensitivity analysis are presented in Table S7. The funnel plot is depicted in Figure S4.

## Discussion

EHRs have been extensively used to document the change of the illness, therapeutic regimens, laboratory test results, and radiological images.^36^ Predicting ADEs based on EHR met the clinical need to reduce ADE rates, and it holds promise for enhancing the quality of healthcare. Owing to the amalgamation of structured and unstructured data, conventional statistical methods face difficulties in predicting adverse events based on EHRs. ML algorithms have been widely embraced in the medical domain, particularly for prognostic predictions, and they are well-suited for intricate data landscapes. Previous systematic reviews emphasized the identification and diagnosis of safety events, and ADE prediction is only part of these studies.^37,38^ In addition, although some articles^5,15,30^ that met the inclusion criteria were published within the search time frame of these systematic reviews, none of them was included. In the present study, we conducted a systematic review of the use of ML algorithms in forecasting multiple drug-related events based on EHRs and clinical notes.

The Stockholm EPR Corpus, MIMIC-IV, and self-built databases were the most commonly used databases. The Stockholm EPR Corpus was developed by Karolinska University Hospital in Sweden, and it encompasses comprehensive diagnostic information, drug administrations, clinical measurements, and free-text clinical notes.^33^ MIMIC-IV is a product of a collaboration between Beth Israel Deaconess Medical Center and the Massachusetts Institute of Technology, and it provides deidentified data, including patient measurements, diagnoses, procedures, treatments, and free-text clinical notes.^39^ One study used a Chinese database covering 30,703 patients (demographic data, procedures, and clinical notes).^34^ Data in the Stockholm EPR Corpus, MIMIC-IV, and the Chinese database were both unstructured and structured,^28,34^ consistent with the actual forecast scenario. In the Chinese database, clinical notes included diagnoses and medications in the Chinese language, and thus, researchers introduced natural language processing (NLP) methods to process them.^34^ Three studies relied on self-built databases derived from the EHRs of hospitals, and they contained structured data.^5,30,35^ Compared with Stockholm EPR, MIMIC-IV, and the Chinese database, the ADE prediction model was built based on a self-built database, allowing researchers to review medical records for more information, thus avoiding missed data. In addition, the occurrence of ADEs in different institutions was inconsistent; hence, the prediction model based on the self-built database was more in line with the actual situation of the institution. International Classification of Disease, Version 10 codes were applied as the standard terminology for diagnoses in studies based on the Stockholm EPR Corpus and MIMIC-IV.^15,28,29,31,32^ The use of standard terminology contributes to greater standardization of the database, thereby enhancing the reliability of the results. Based on these results, we recommend applying self-built databases and standard terminology to build ADE prediction models and improve the applicability and accuracy of the model.

ML is the scientific discipline that focuses on how computers learn from data, and it arose at the intersection of statistics.^40^ This marriage between statistics and computer science is driven by the unique computational challenges of building statistical models from massive datasets.^41^ The types of ML are conveniently subclassified into the categories supervised learning and unsupervised learning. Because it is based on existing data, supervised learning was used to build the ADE prediction model. Twenty ML algorithms were employed in the included studies, with ensemble learning emerging as the predominant model. Ensemble learning encompassing predictions from multiple weak learners can obtain superior predictions.^42,43^ Bagging and boosting algorithms are the representative ensemble learning methods. In boosting algorithms, each weak learner undergoes further training with an updated set including the misclassified instances from the prior training iteration.^44^ Five studies documented the performances of boosting algorithms, with aggregate performance metrics portraying favorable outcomes.^5,15,30,32,34^ The average AUC and precision of boosting algorithms were 0.82 (95% CI = 0.72–0.92) and 0.47 (95% CI = 0.10–0.69). AdaBoost and XGBoost demonstrated superior performance compared with LightGBM, gradient boosting machine, and gradient boosting decision tree, as evidenced by the AUC, F1 scores, and precision, although meta-regression was infeasible because of the inadequate contingency tables.

Bagging algorithms, involving the aggregation of predictions from multiple decision trees, delineate a distinct approach from boosting algorithms. This method entails the resampling of data from the training set with equivalent cardinality to the original set, consequently mitigating classifier variance and overfitting.^45^ RF, as the representative algorithm, was the most frequently reported.^5,15,28–30,32–35^ EHR data featuring numerous sparse features can lead to suboptimal predictive performance for RF.^46^ Therefore, the RF algorithms in the included studies were refined through adjustments in configuration, learned weights, tree sizes, and integration with diverse resampling approaches. The results indicated that the mean AUC and precision for unimproved RF were 0.81 (95% CI = 0.74–0.94) and 0.36 (95% CI = 0.10–0.75), whereas those for improved RF were 0.83 (95% CI = 0.75–0.95) and 0.51 (0.49–0.53). Among these, improved RF combined with resampling until an informative feature was found or until no more features were left demonstrated superior performance, yielding an AUC of 0.95 and an F1 score of 0.89.^28^

SVM was also widely reported. Its fundamental principle involves identifying the maximum margin hyperplane within the input space to segregate the training dataset.^47^ SVM excels in addressing pattern recognition challenges associated with limited samples, non-linearity, and high dimensionality, particularly in the realm of classification problems.^48^ Therefore, the performance of SVM was inferior to that of ensemble learning, with a mean AUC of only 0.63 (95% CI = 0.59–0.67).^29,32,34^ According to the aforementioned ML results, ensemble learning might be more popular and more suitable for building ADE prediction models. In addition, we encourage adjustment of the configuration to improve model performance.

Several studies described findings on the performance of LR. It was found to yield comparable results to non-LR methods, such as SVM, artificial neural network, K-nearest neighbor, and naïve Bayes (NB).^15,32,34^ Meta-regression analysis indicated that non-LR methods exhibited higher specificity than LR, albeit with lower sensitivity. Therefore, LR might also achieve favorable performance. The selection of appropriate algorithms should be contingent on the specific research issue and application context.

Eight analyses provided information on the types of ADEs.^5,15,28–33^ Allergies were most frequently mentioned, with an incidence ranging from 1% to 6%. Drug allergies can be linked to any form of medication. Although most allergies are transitory, some can result in severe consequences, such as drug reaction with eosinophilia and systemic symptoms and SJS.^49^ Oversedation\hypotension were also prevalent and associated with blood pressure medications, sedative hypnotics, and anesthetics, and its incidence rates ranged from 0.3% to 2%. This incidence might be higher in pediatric inpatients^30^ because of the considerable individual variability in sedative hypnotics or anesthetics dosages among children coupled with significant variations in children’s sensitivity to these drugs.^50^ Furthermore, respiratory depression, bronchospasm, and dyspnea were exclusively observed in children, and they might be linked to the use of sedative hypnotics or anesthetics, underscoring the need for cautious administration of these medications in pediatric patients.

The risk factors associated with ADEs were reported in four studies.^5,15,30,34^ Although these risk factors exhibited variations, certain common factors consistently emerged, including the length of hospitalization, polypharmacy, patient age, and the use of high-risk medications.^5,15,30,34^ Cross-sectional investigations demonstrated that patients requiring prolonged hospitalization and a higher number of medications have a higher risk of drug-related injuries.^13,51,52^ Advanced age emerged as a significant risk factor for ADEs given that older patients are more susceptible to drug-related events because of the presence of multiple comorbidities, polypharmacy, challenges in medication monitoring, and age-related changes in pharmacokinetics and pharmacodynamics.^53^ High-risk medications, including glucocorticoids, anticoagulants, non-steroidal anti-inflammatory drugs, and chemotherapeutic agents, were also among the risk factors.^54^ Therefore, patients undergoing surgery usually only receive intravenous infusion therapy during hospitalization, and they rarely experience ADEs. These findings underscored the importance of reducing hospital stays, simplifying treatment regimens, and avoiding the use of high-risk medications as potential strategies for ADE prevention. Although it is difficult to avoid these risk factors in clinical practice, healthcare providers are advised to exercise heightened vigilance in monitoring patients with risk factors and to promptly address ADEs when they occur.

The heterogeneity among the included studies was significant, and although it was reduced in the same study, it was not eliminated. The high heterogeneity was related to differences in the databases used, predictors, ML algorithms, hyperparameters, and the populations included, making it difficult to avoid.^55^

The present study had several limitations. First, the quality of the included studies was not high. Only three studies were deemed to have a low risk of bias and scores higher than 25 (maximum, 50) based on PROBAST and ChAMAI assessments. Most of the included studies lacked proper reporting of data and data preprocessing procedures. Second, contingency tables, which could provide a pooled estimate for comparing predictive performance, were only available for three studies involving 13 models. Third, certain input variables, such as chief complaints, can benefit from advanced preprocessing methods such as NLP. Unfortunately, only one study combined NLP with ML for ADE prediction, but the reliability of this method was not verified. Finally, the application of new ML models, such as convolutional neural networks, recurrent neural networks, and bidirectional long short-term memory with conditional random field algorithms, for predicting drug safety events using EHRs was rare. Hence, researchers should focus on innovative ML algorithms to enhance the predictive capabilities of models and promote the application of these advancements in the future.

## Conclusions

This systematic review provided evidence supporting the potential of ML to be incorporated into EHRs for predicting multiple ADEs, improving the quality of patient care, and reducing drug-related harm. Future studies should consider adopting more rigorous reporting standards and newer ML techniques to enhance the effectiveness of ML models in clinical practice.

## Supplemental Material

sj-pdf-1-imr-10.1177_03000605241302304 - Supplemental material for Machine learning to predict adverse drug events based on electronic health records: a systematic review and meta-analysis Supplemental material, sj-pdf-1-imr-10.1177_03000605241302304 for Machine learning to predict adverse drug events based on electronic health records: a systematic review and meta-analysis by Qiaozhi Hu, Jiafeng Li, Xiaoqi Li, Dan Zou, Ting Xu and Zhiyao He in Journal of International Medical Research
