# Supplementary material for: Machine learning to predict adverse drug events based on electronic health records: a systematic review and meta-analysis
Source: J Int Med Res. 2024 Dec 13;52(12):03000605241302304. doi: 10.1177/03000605241302304 (PMC11639029; doi:10.1177/03000605241302304)
Supplement: sj-pdf-1-imr-10.1177_03000605241302304 - Supplemental material for Machine learning to predict adverse drug events based on electronic health records: a systematic review and meta-analysis [file sj-pdf-1-imr-10.1177_03000605241302304.pdf]

Supplement Table 1. PRISMA Checklist. PRISMA checklist for this network meta-analysis

Section and Topic	Item #	Checklist item	Location where item is reported
TITLE			
Title	1	Identify the report as a systematic review.	1
ABSTRACT			
Abstract	2	See the PRISMA 2020 for Abstracts checklist.	2-3
INTRODUCTION			
Rationale	3	Describe the rationale for the review in the context of existing knowledge.	3-4
Objectives	4	Provide an explicit statement of the objective(s) or question(s) the review addresses.	4
METHODS			
Eligibility criteria	5	Specify the inclusion and exclusion criteria for the review and how studies were grouped for the syntheses.	4
Information sources	6	Specify all databases, registers, websites, organisations, reference lists and other sources searched or consulted to identify studies. Specify the date when each source was last searched or consulted.	4
Search strategy	7	Present the full search strategies for all databases, registers and websites, including any filters and limits used.	4
Selection process	8	Specify the methods used to decide whether a study met the inclusion criteria of the review, including how many reviewers screened each record and each report retrieved, whether they worked independently, and if applicable, details of automation tools used in the process.	4
Data collection process	9	Specify the methods used to collect data from reports, including how many reviewers collected data from each report, whether they worked independently, any processes for obtaining or confirming data from study investigators, and if applicable, details of automation tools used in the process.	4
Data items	10a	List and define all outcomes for which data were sought. Specify whether all results that were compatible with each outcome domain in each study were sought (e.g. for all measures, time points, analyses), and if not, the methods used to decide which results to collect.	4
	10b	List and define all other variables for which data were sought (e.g. participant and intervention characteristics, funding sources). Describe any assumptions made about any missing or unclear information.	4
Study risk of bias assessment	11	Specify the methods used to assess risk of bias in the included studies, including details of the tool(s) used, how many reviewers assessed each study and whether they worked independently, and if applicable, details of automation tools used in the process.	4-5

Section and Topic	Item #	Checklist item	Location where item is reported
Effect measures	12	Specify for each outcome the effect measure(s) (e.g. risk ratio, mean difference) used in the synthesis or presentation of results.	5
Synthesis methods	13a	Describe the processes used to decide which studies were eligible for each synthesis (e.g. tabulating the study intervention characteristics and comparing against the planned groups for each synthesis (item #5)).	5
	13b	Describe any methods required to prepare the data for presentation or synthesis, such as handling of missing summary statistics, or data conversions.	5
	13c	Describe any methods used to tabulate or visually display results of individual studies and syntheses.	5
	13d	Describe any methods used to synthesize results and provide a rationale for the choice(s). If meta-analysis was performed, describe the model(s), method(s) to identify the presence and extent of statistical heterogeneity, and software package(s) used.	5
	13e	Describe any methods used to explore possible causes of heterogeneity among study results (e.g. subgroup analysis, meta-regression).	5
	13f	Describe any sensitivity analyses conducted to assess robustness of the synthesized results.	5
Reporting bias assessment	14	Describe any methods used to assess risk of bias due to missing results in a synthesis (arising from reporting biases).	5
Certainty assessment	15	Describe any methods used to assess certainty (or confidence) in the body of evidence for an outcome.	5
RESULTS			
Study selection	16a	Describe the results of the search and selection process, from the number of records identified in the search to the number of studies included in the review, ideally using a flow diagram.	5-6
	16b	Cite studies that might appear to meet the inclusion criteria, but which were excluded, and explain why they were excluded.	5-6
Study characteristics	17	Cite each included study and present its characteristics.	6, table1
Risk of bias in studies	18	Present assessments of risk of bias for each included study.	6
Results of individual studies	19	For all outcomes, present, for each study: (a) summary statistics for each group (where appropriate) and (b) an effect estimate and its precision (e.g. confidence/credible interval), ideally using structured tables or plots.	6
Results of syntheses	20a	For each synthesis, briefly summarise the characteristics and risk of bias among contributing studies.	7
	20b	Present results of all statistical syntheses conducted. If meta-analysis was done, present for each the summary estimate and its precision (e.g. confidence/credible interval) and measures of statistical heterogeneity. If comparing groups, describe the direction of the effect.	7
	20c	Present results of all investigations of possible causes of heterogeneity among study results.	7

Section and Topic	Item #	Checklist item	Location where item is reported
	20d	Present results of all sensitivity analyses conducted to assess the robustness of the synthesized results.	7
Reporting biases	21	Present assessments of risk of bias due to missing results (arising from reporting biases) for each synthesis assessed.	7-8
Certainty of evidence	22	Present assessments of certainty (or confidence) in the body of evidence for each outcome assessed.	7
DISCUSSION			
Discussion	23a	Provide a general interpretation of the results in the context of other evidence.	8-10
	23b	Discuss any limitations of the evidence included in the review.	11
	23c	Discuss any limitations of the review processes used.	11
	23d	Discuss implications of the results for practice, policy, and future research.	11
OTHER INFORMATION			
Registration and protocol	24a	Provide registration information for the review, including register name and registration number, or state that the review was not registered.	4
	24b	Indicate where the review protocol can be accessed, or state that a protocol was not prepared.	4
	24c	Describe and explain any amendments to information provided at registration or in the protocol.	4
Support	25	Describe sources of financial or non-financial support for the review, and the role of the funders or sponsors in the review.	12
Competing interests	26	Declare any competing interests of review authors.	12
Availability of data, code and other materials	27	Report which of the following are publicly available and where they can be found: template data collection forms; data extracted from included studies; data used for all analyses; analytic code; any other materials used in the review.	12

Supplement Table 2. Search Strategy

Database	Search Strategy
PubMed	(((machine learn [mh]) OR (machine learn [tw])) OR ((machine learning [mh]) OR (machine learning [tw])) OR ((deep learning [mh]) OR (deep learning [tw])) OR ((artificial intelligence [mh]) OR (artificial intelligence [tw])) OR ((artificial learning [mh]) OR (artificial learning [tw])) OR ((machine intelligence [mh]) OR (machine intelligence [tw])) OR ((neural networks [mh]) OR (neural networks [tw]))) AND (((adverse drug reaction [mh]) OR (adverse drug reaction [tw])) OR ((adverse drug event [mh]) OR (adverse drug event [tw])) OR ((ADE [mh]) OR (ADE [tw])) OR ((ADR [mh]) OR (ADR [tw]))) AND (((predict[mh]) OR (predict[tw])) OR ((prediction[mh]) OR (prediction [tw])) OR ((predicting[mh]) OR (predicting [tw])) OR ((risk [mh]) OR (risk [tw])) OR ((predictive [mh]) OR (predictive [tw])))
Embase	(machine learn.mp. or exp machine learn/ or machine learning.mp. or exp machine learning/ or deep learning.mp. or exp deep learning/ or artificial intelligence.mp. or exp artificial intelligence/ or artificial learning.mp. or exp artificial learning/ or machine intelligence.mp. or exp machine intelligence/ or neural networks.mp. or exp neural networks/) AND (exp adverse drug reaction/ or adverse drug reaction. mp. or exp adverse drug event/ or adverse drug event. mp. or exp ADE/ or ADE.mp. or exp ADR/ or ADR.mp.) AND (exp predict/ or predict. mp. or exp predicting/ or predicting. mp. or exp prediction/ or prediction.mp. or exp risk/ or risk.mp. or exp predictive / or predictive.mp. or exp predictive/ or predictive.mp.)
Web of Science	TS= (machine learn OR machine learning OR deep learning OR artificial intelligence OR artificial learning OR machine intelligence OR neural networks) AND TS= (adverse drug reaction OR adverse drug event OR ADE OR ADR) AND TS= (predict OR prediction OR predicting OR risk OR predictive OR predictive)
IEEE	(machine learn OR machine learning OR deep learning OR artificial intelligence OR artificial learning OR machine intelligence OR neural networks) [Abstract] AND (adverse drug reaction OR adverse drug event OR ADE OR ADR) [Abstract] AND (predict OR prediction OR predicting OR risk OR predictive OR predictive) [Abstract]

Supplement Table 3. Quality assessment based on PROBAST

Study ID	Risk of bias				Applicability concerns			Overall	
	Participant selection	Predictors	Outcome	Analysis	Participant selection	Predictors	Outcome	Risk of Bias	Applicability concerns
Hu, 2022[5]	low	low	low	low	low	low	low	low	low
Yu, 2021 [27]	low	low	low	low	low	low	low	low	low
Langenberger, 2023[15]	low	low	low	low	low	low	low	low	low
Karlsson, 2014[25]	high	high	unclear	low	low	high	unclear	high	high
Ponraj, 2021[32]	high	unclear	low	high	high	high	high	high	high
Karlsson, 2016[26]	low	high	low	high	low	low	low	high	low
Zhao, 2021[31]	low	low	low	high	low	low	low	high	low
Zhao, 2015a[29]	low	high	low	high	low	low	low	high	low
Zhao, 2015b[30]	low	high	low	high	low	low	low	high	low
Zhao, 2016[28]	low	high	low	high	low	low	low	high	low

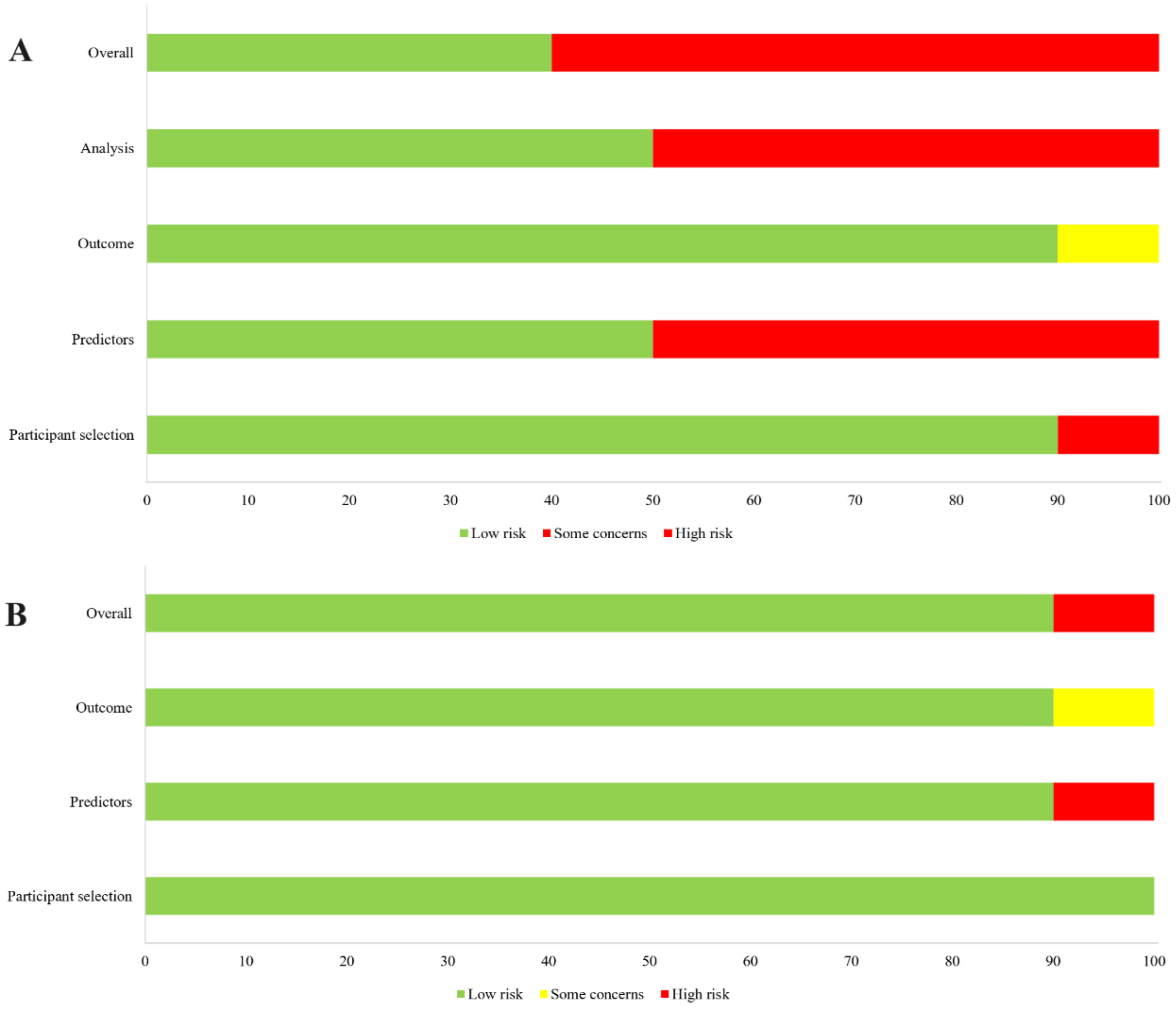

Supplement Figure 1 The evaluation result diagram of PROBAST A, risk of bias; B, Applicability

Supplement Table 4. Quality assessment based on the ChAMAI checklist

Study ID	Problem understanding (10)	Data understanding (6)	Data preparation (8)	Modeling (6)	Validation (12)	Deployment (8)	Total (50)
Hu, 2022[5]	9.5	5	0	6	4	3	27.5
Yu, 2021 [27]	9	5	0	6	2.5	2.5	25
Langenberger, 2023[15]	4.5	5	4	6	10	3	32.5
Karlsson, 2014[25]	6	1	0	6	10	1	24
Ponraj, 2021[32]	4	2	2	6	5	1	20
Karlsson, 2016[26]	6	1	0	6	8	1	21
Zhao, 2021[31]	6	2	3	6	5	1	23
Zhao, 2015a[29]	6	1	0	6	9	1	23
Zhao, 2015b[30]	6	1	0	6	7	0	20
Zhao, 2016[28]	6	1	0	6	7	0	20
Hu, 2022[5]	6.3	2.4	0.9	6	6.75	1.35	23.7

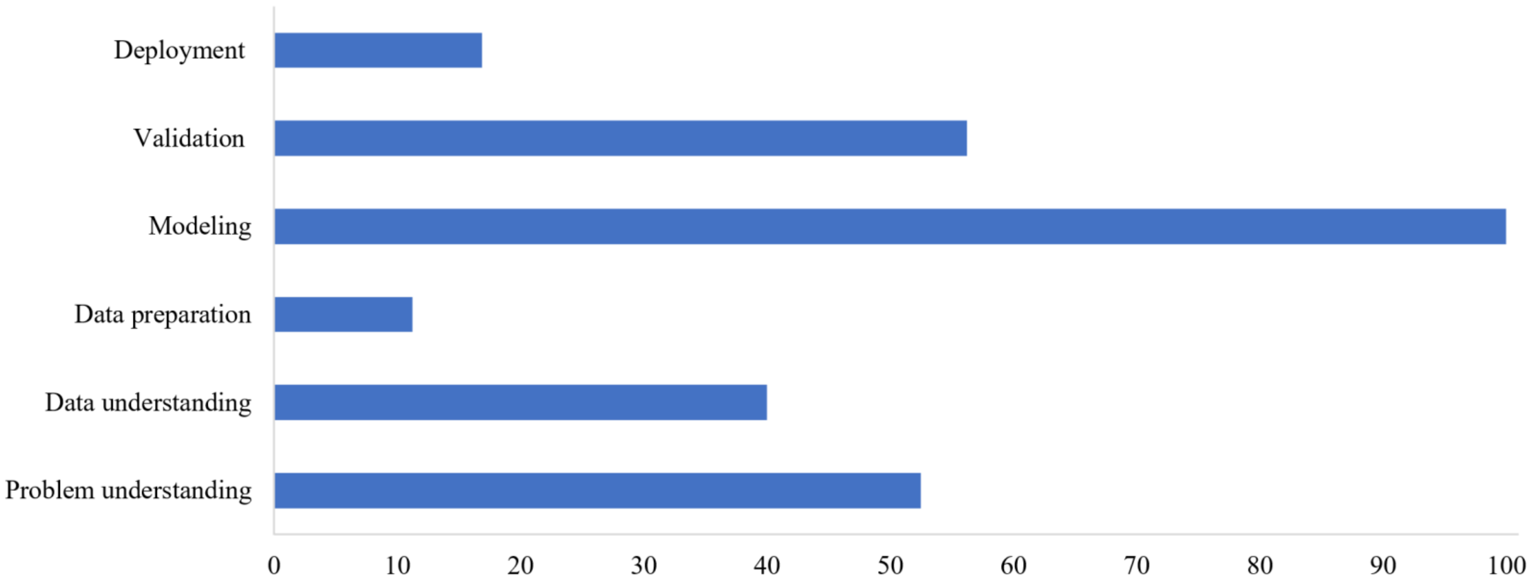

Supplement Figure 2 The scoring rate of ChAMAI checklist

Supplement Table 5 ADR/ADE involved in included studies

Study ID	ADE	Number of ADE
Hu, 2022[5]	Hypokalemia, Hyperkalemia, Hypoglycemia, Hepatotoxicity/Transaminase disorder, Nephrotoxicity/ Creatinine disorder, Urinary Retention, Candidiasis, Infection of the upper respiratory tract, Myalgia, Allergy, Fever, Weakness, Pain, Cold sweating, Dizziness, Sleepiness, Tremor, Constipation, Diarrhea, Nausea, Anorexia, Vomiting, Acute gastric mucosal injury, Abdominal distension, Hypotension, Palpitation, Bradycardia, Vascular headache, Hemorrhage, Leukopenia, Thrombocytopenia, Hemoglobin decline	32
Yu, 2021 [27]	Diarrhea, Constipation, Vomiting, Convulsions, Convulsions grandmal, Over sedation\Hypotension, Rash, Candidiasis, Infection fungal, Hyperkalemia, Hypokalemia, Hypoglycemia, Hyperglycemia, Hyponatremia, Hepatotoxicity/Increased transaminases, Coagulopathy, Respiratory depression, Bronchospasm, Dyspnea, Leukopenia, Dystonia, Arthritis, Allergic reactions, Anaphylactoid reaction, Nephritis/Nephrosis, Euphoria, Tachycardia	27
Langenberger, 2023[15]	Other secondary thrombocytopenia, Other iatrogenic hypotension, Fever, unspecified, Dermatitis due to drugs and medicines taken internally, Dermatitis due to drugs and medicines taken internally, Enterocolitis due to Clostridium difficile, Hypotension due to drugs, Essential and other specified forms of tremor, Toxic gastroenteritis and colitis, Generalized skin eruption due to drugs and medicaments taken internally, Nephropathy induced by other drugs, medicaments and biological substances, Hepatitis, unspecified, Drug-induced polyneuropathy, Polyneuropathy due to drugs, Drug-induced adrenocortical insufficiency, Autoimmune hemolytic anemias, Secondary cardiomyopathy, unspecified, Localized skin eruption due to drugs and medicaments taken internally, Drug induced fever, Postprocedural (acute) (chronic) kidney failure, Other specified hypoglycemia, Drug withdrawal syndrome in newborn, Drug withdrawal syndrome in newborn, Other iatrogenic hypothyroidism, Cardiomyopathy due to drug and external agent, Cushing's syndrome, Drug-induced interstitial lung disorders, unspecified, Iatrogenic pituitary disorders, Hypothyroidism due to medicaments and other exogenous substances, Drug-induced tremor, Drug-induced myopathy, Neonatal withdrawal symptoms from maternal use of drugs of addiction, Allergic purpura, Folate-deficiency anemia, Contact dermatitis and other eczema due to drugs and medicines in contact with skin, Toxic liver disease with acute hepatitis, Newborn affected by other maternal medication, Drug-induced hypoglycemia without coma, Toxic liver disease, unspecified, Toxic liver disease with cholestasis, Iodine hypothyroidism, Stevens-Johnson syndrome, Other nonthrombocytopenic purpura, Abuse of laxatives, Other nonthrombocytopenic purpuras<br>Toxic liver disease with hepatitis, not elsewhere classified, Toxic liver disease with chronic persistent hepatitis, Drug-induced Cushing's syndrome, Abuse of other non-psychoactive substances, Drug-induced nonautoimmune hemolytic anemia, Other chorea's, Analgesic nephropathy, Neuroleptic malignant syndrome, Acute drug-induced interstitial lung disorders, Chronic drug-induced interstitial lung disorders, Erythema multiforme, unspecified, Toxic liver disease with other disorders of liver, Drug induced acute pancreatitis without necrosis or infection, Nephropathy induced by unspecified drug, medicament or biological substance, Unspecified misadventure during medical care, Erythema multiforme, unspecified, Abuse of steroids or hormones, Toxic liver disease with fibrosis and cirrhosis of liver, Allergic contact dermatitis due to drugs in contact with skin, Maternal anesthesia and analgesia affecting fetus or newborn, Drug-induced thyroiditis, Unspecified contact dermatitis due to drugs in contact with skin, Newborn affected by maternal anesthesia and analgesia in pregnancy, labor and delivery, Sideroblastic anemia, Malignant neuroleptic syndrome, Other erythema multiforme, Unspecified misadventure during surgical and medical care, Acute dermatitis due to solar radiation, Acute dermatitis due to solar radiation, Acute dermatitis due to solar radiation, Drug-induced autoimmune hemolytic anemia, Erythema multiforme major, Iatrogenic thyroiditis, Drug-induced systemic lupus erythematosus, Anticonvulsants affecting fetus or newborn via placenta or breast milk, Osteonecrosis due to drugs, unspecified bone, Toxic nephropathy, not elsewhere classified, Antimetabolic agents affecting fetus or	102

	newborn via placenta or breast milk, Withdrawal symptoms from therapeutic use of drugs in newborn, Drug-induced hypopituitarism, Secondary parkinsonism due to other external agents, Drug induced acute pancreatitis with uninfected necrosis, Irritant contact dermatitis due to drugs in contact with skin, Drug-induced gout, unspecified site, Drug-induced gout, multiple sites, Cardiac complications of anesthesia or other sedation in labor and delivery, delivered, with or without mention of antepartum condition, Drug-induced folate deficiency anemia, Hypoglycemic coma, Abuse of herbal or folk remedies, Drug-induced chorea, Dermatitis due to other specified substances taken internally, Drug phototoxic response, Systemic sclerosis induced by drug and chemical, Nephropathy induced by heavy metals, Cardiac complications of anesthesia or other sedation in labor and delivery, delivered, with mention of postpartum complication, Central nervous system complications of anesthesia during labor and delivery, Central nervous system complications of anesthesia or other sedation in labor and delivery, delivered, with mention of postpartum complication	
Karlsson, 2014[25]	Drug-induced aplastic anemia, secondary sideroblastic anemia due to drugs and toxins, mental and behavioral disorders due to opioid use (acute intoxication), mental and behavioral disorders due to opioid use (chronic intoxication), Drug use), cardiomyopathy due to drugs and other external factors, dermatitis due to ingestion, kidney disease due to other drugs, pharmaceutical and biological agents, maternal care (suspected) of fetal injury through drugs, anaphylactic shock not specified, angioneurotic edema, allergies not specified, Other complications associated with infusion, transfusion, and therapeutic injection, anaphylactic shock due to side effects of the correct use of the correct drug or agent, side effects of an unspecified drug or agent	14
Ponraj, 2021[32]	N	N
Karlsson, 2016[26]	Secondary sideroblastic anemia due to drugs and toxins, Drug-induced adrenocortical insufficiency, Mental and behavioral disorders (MBOs) due to use of opioids: acute intoxication, MBDs due to use of opioids: dependence syndrome, MBDs due to use of sedatives or hypnotics: acute intoxication, MBOs due to use of sedatives or hypnotics: dependence syndrome, MBDs due to use of other stimulants, including caffeine: acute intoxication, MBOs due to use of other stimulants, including caffeine: harmful use, MHOs due to use of other stimulants, including caffeine: dependence syndrome, MHOs due to multiple drug use: acute intoxication, MBDs due to multiple drug use: dependence syndrome, MBDs due to multiple drug use: unspecified mental and behavioral disorder, Drug Induced Dystonia, Drug-induced headache, not elsewhere classified, Drug-Induced Polyneuropathy, Cardiomyopathy Due To Drug And External Agent, Hypotension Due To Drugs, Generalized skin eruption due to drugs and medicaments, Localized skin eruption due to drugs and medicaments, Maternal care for (suspected)damage to fetus by drugs, Toxic effect of unspecified gases, fumes and vapors, Adverse effects: anaphylactic shock, unspecified, Adverse effects: angioneurotic oedema, Adverse effects: allergy, unspecified, Other complications following infusion, transfusion and therapeutic injection, Anaphylactic shock due to correct drug or medicament properly administered, Unspecified adverse effect of drug or medicament	27
Zhao, 2021[31]	N	N
Zhao, 2015a[29]	Secondary sideroblastic anemia due to drugs and toxins, Drug-induced adrenocortical insufficiency, Mental and behavioral disorders (MBDs) due to use of opioids: acute intoxication, MBDs due to use of opioids: dependence syndrome, MBDs due to use of sedatives or hypnotics: acute intoxication, MBDs due to use of sedatives or hypnotics: dependence syndrome, MBDs due to use of other stimulants, including caffeine: acute intoxication, MBDs due to use of other stimulants, including caffeine: harmful use, MBDs due to use of other stimulants, including caffeine: dependence syndrome, MBDs due to multiple drug use: acute intoxication, MBDs due to multiple drug use: dependence syndrome, MBDs due to multiple drug use: unspecified mental and behavioral disorder, Drug-induced dystonia, Drug-induced tremor, Drug-induced headache, not elsewhere classified, Drug-induced polyneuropathy, Cardiomyopathy due to drugs and other external agents, Hypotension due to drugs, Generalized skin eruption due to drugs and medicaments, Localized skin eruption due to drugs and medicaments, Maternal care for (suspected) damage	27

	to fetus by drugs, Adverse effects: anaphylactic shock, unspecified, Adverse effects: angioneurotic oedema, Adverse effects: allergy, unspecified, Other complications following infusion, transfusion and therapeutic injection, Anaphylactic shock due to correct drug or medicament properly administered, Unspecified adverse effect of drug or medicament	
Zhao, 2015b[30]	Secondary sideroblastic anemia due to drugs and toxins, Drug-induced dystonia, Drug-induced headache, not elsewhere classified, Drug-induced polyneuropathy, Hypotension due to drugs, Generalized skin eruption due to drugs and medicaments, Localized skin eruption due to drugs and medicaments, Maternal care for (suspected) damage to fetus by drugs, Adverse effects: anaphylactic shock, unspecified, Adverse effects: angioneurotic oedema, Adverse effects: allergy, unspecified, Other complications following infusion, transfusion and therapeutic injection, Anaphylactic shock due to correct drug or medicament properly administered, Unspecifed adverse effect of drug or medicament	14
Zhao, 2016[28]	Drug-induced aplastic anemia, Drug-induced secondary sideroblastic anemia, Secondary thrombocytopenia, Drug-induced adrenocortical insufficiency, Drug-induced polyneuropathy, Drug-induced hypotension, Drug-induced generalized skin eruption, Drug-induced localized skin eruption, Drug-induced osteoporosis with pathological fracture, Drug-induced osteoporosis, Maternal care for damage to fetus by drugs, Drug-induced fever, Adverse effects: anaphylactic shock, Adverse effects: angioneurotic oedema, Adverse effects: allergy, Vascular complications following infusion, transfusion and therapeutic injection, Other complications following infusion, transfusion and therapeutic injection, Drug-induced anaphylactic shock, Unspecified adverse effect of drug or medicament	19

Supplement Table 6. Contingency tables for adverse drug reaction or event prediction (20 tables from 3 studies)

Study ID	Model	TP	FP	FN	TN	Accuracy	Precision	Sensitivity(recall)	Specificity	F1 score
Hu, 2022[5]	GBDT	27	19	29	285	86.67%	58.69%	48.21%	93.75%	52.94%
Hu, 2022[5]	LightGBM	23	16	33	288	86.39%	58.97%	41.07%	94.74%	48.42%
Hu, 2022[5]	Adaboost	24	11	32	293	88.06%	68.57%	42.86%	96.38%	52.75%
Hu, 2022[5]	RF	18	6	38	298	87.78%	75.00%	32.14%	98.06%	45.00%
Hu, 2022[5]	Catboost	25	16	31	288	86.94%	60.97%	44.64%	94.74%	51.55%
Hu, 2022[5]	TOPT	27	15	29	289	87.78%	64.29%	48.21%	95.07%	55.10%
Hu, 2022[5]	XGboost	25	14	31	290	87.50%	64.10%	44.64%	95.40%	52.63%
Yu, 2021 [27]	GBDT	11	14	33	292	86.57%	44.00%	25.00%	95.43%	31.88%
Yu, 2021 [27]	LightGBM	3	8	41	298	86.00%	27.27%	6.82%	97.39%	10.91%
Yu, 2021 [27]	Adaboost	7	10	37	296	86.57%	41.18%	15.91%	96.73%	22.95%
Yu, 2021 [27]	RF	6	20	38	286	83.43%	23.08%	13.64%	93.46%	17.14%
Yu, 2021 [27]	Catboost	6	7	38	299	87.14%	46.15%	13.64%	97.71%	21.05%
Yu, 2021 [27]	TOPT	6	2	38	304	88.57%	75.00%	13.64%	99.35%	23.08%
Yu, 2021 [27]	XGboost	9	17	35	289	85.14%	34.61%	20.45%	94.44%	25.71%
Langenberger, 2023[15]	RF	1591	13419	628	26399	63.80%	9.60%	71.70%	66.30%	17.00%
Langenberger, 2023[15]	GBM	1498	13332	721	26486	66.70%	10.10%	67.50%	66.520%	17.60%
Langenberger, 2023[15]	LASSO	1518	13816	701	26002	65.50%	9.70%	68.40%	65.30%	17.00%
Langenberger, 2023[15]	Ridge	1502	13824	717	25994	66.10%	9.80%	67.70%	65.280%	17.10%
Langenberger, 2023[15]	EN	1513	13925	706	25893	66.10%	9.80%	68.20%	65.030%	17.20%
Langenberger, 2023[15]	LR	1498	13788	721	26030	66.10%	9.80%	67.50%	65.370%	17.10%

TP, true-positive; FP, false-positive; TN, true-negative; FN, false-negative

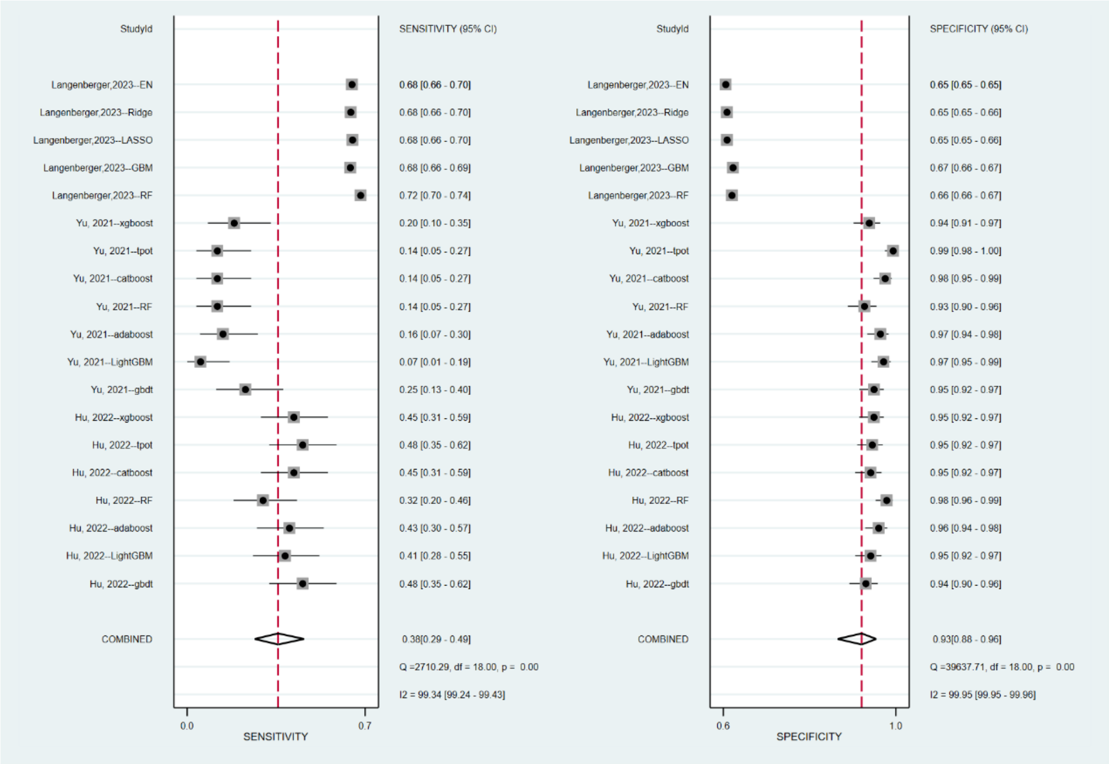

Supplement Figure 3. Forest plots of sensitivity and specificity (LR excepted)

Supplement Table 7. Subgroup analysis according to different population

Population	Included studies	Pooled specificity (95% CI)	I^2	Pooled sensitivity (95% CI)	I^2	Pooled AUCPRC (95% CI)
Older inpatients	Hu, 2022[5]	96% (94-96)	25.74	43% (38-39)	0.00	80% (76-83)
Pediatric inpatients	Yu, 2021 [27]	97% (95-98)	72.83	15% (11-20)	11.55	41% (36-45)
All population	Langenberger, 2023[15]	65% (65-66)	87.58	69% (67-70)	67.12	71% (67-75)

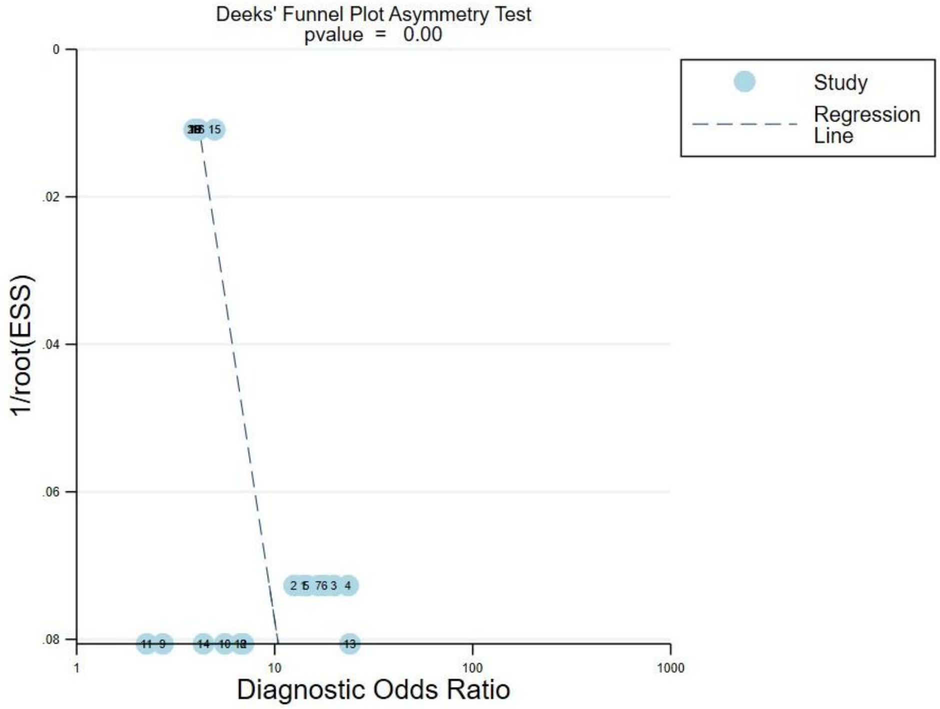

Supplement Figure 4. Publication bias for adverse drug reaction or event prediction (20 tables from 3 studies)
